# Epigenetic Mechanisms beyond Tumour–Stroma Crosstalk

**DOI:** 10.3390/cancers13040914

**Published:** 2021-02-22

**Authors:** Teresa Gagliano, Claudio Brancolini

**Affiliations:** Department of Medicine, University of Udine, 33100 Udine, Italy; claudio.brancolini@uniud.it

**Keywords:** microenvironment, epigenetics, cell–cell interaction

## Abstract

**Simple Summary:**

In this review article, we will deepen the topic of cancer cell communication with the surrounding non-cancerous cells. In particular, the non-mutational events that modified gene expression, namely “epigenetics”, involved in cell–cell interaction will be the center of this work. Many studies have described the mechanism of back-and-forth communication between cancer and stromal cells, and very recent studies suggested that epigenetics may play an important role in this setting. This work will describe recent advances in the field of epigenetic mechanisms involved in tumour–stroma crosstalk. Understanding these processes can be useful in future cancer therapy design.

**Abstract:**

Despite cancer having been usually considered the result of genetic mutations, it is now well established that epigenetic dysregulations play pivotal roles in cancer onset and progression. Hence, inactivation of tumour suppressor genes can be gained not only by genetic mutations, but also by epigenetic mechanisms such as DNA methylation and histone modifications. To occur, epigenetic events need to be triggered by genetic alterations of the epigenetic regulators, or they can be mediated by intracellular and extracellular stimuli. In this last setting, the tumour microenvironment (TME) plays a fundamental role. Therefore, to decipher how epigenetic changes are associated with TME is a challenge still open. The complex signalling between tumour cells and stroma is currently under intensive investigation, and most of the molecules and pathways involved still need to be identified. Neoplastic initiation and development are likely to involve a back-and-forth crosstalk among cancer and stroma cells. An increasing number of studies have highlighted that the cancer epigenome can be influenced by tumour microenvironment and vice versa. Here, we discuss about the recent literature on tumour–stroma interactions that focus on epigenetic mechanisms and the reciprocal regulation between cancer and TME cells.

## 1. Introduction

To ensure genome stability, DNA accessibility has to be sophisticatedly regulated. Epigenetics consist of chemical modifications of DNA and chromatin which, in turn, regulate gene expression. Genetics, environmental and metabolic stimuli can unsettle the homeostasis of chromatin by determining its restrictive or permissive abnormal status. During cancer cell proliferation, the setting of epigenetic changes is maintained across cell division through further epigenetic mechanisms or gene regulator loops that sustain adaptive clones, thus stimulating cancer progression [1].

Defects in chromatin regulators have been detected in both solid and blood cancers, and the importance of epigenetics in cancer cells has been demonstrated by several groups in different cancer types [2,3,4].

**Figure 1:**
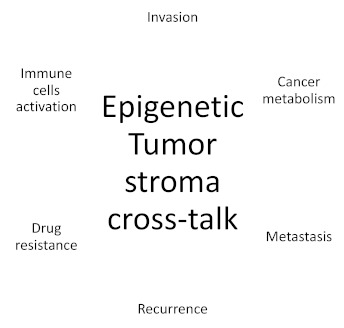
Mechanisms regulated by epigenetic tumour–stroma crosstalk.

During the last decades, cancer has been identified as a multifaceted system which can surpass normal healthy tissue complexity. For long almost completely neglected by researchers, all non-tumoural cells interfacing with cancer cells, defined as the tumour microenvironment (TME), have now a prominent role in cancer research. TME is composed of several cell types including endothelial cells, pericytes, fibroblasts, lymphocytes and macrophages [5]. Many studies have reported a pivotal role for TME in cancer onset, progression and behavior. Stromal cells can influence cancer cells at different levels by promoting invasion, metastasis and resistance to treatment [6,7,8] (Figure 1).

However, the molecular mechanisms governing cancer cells/TME communication are largely unknown and relatively under-investigated. It is highly unlikely, within cell communication, that genetic mutations can occur due to cell–cell interaction; thus, epigenetic resetting may be the primary way of communication between cancer and stroma cells. Not surprisingly, many recent studies have highlighted the contribution of the epigenetic mechanisms in the regulation of tumour–stroma crosstalk. Neoplastic cells exploit different strategies to reprogram the epigenetic landscape of the microenvironment. Their actions can be direct, through the dysregulation of the epigenetic regulators, or indirect, through the modulation of signalling pathways that control the activity of epigenetic regulators. These strategies involve the classical arsenal of epigenetic actions including histones acetylation and methylation, DNA methylation, long noncoding RNAs and even RNA editing (summarized in Table 1). In this review we aimed to recapitulate all the recent advancements in the field and to analyse future prospects which can, eventually, have a prominent role in future therapy design.

## 2. Tumour–Stroma Crosstalk Mediated by Epigenetic Mechanisms

Both haematological and solid tumours are epigenetically influenced by TME. However, in this review we will be focusing on solid cancers and, in particular, breast, ovarian, colorectal and pancreatic cancers, for which, recently, epigenetic mechanisms have been intensively investigated.

### 2.1. Breast Cancer

Breast cancer (BC) is among the most malignant diseases in women [9]. BC is a very heterogenous disease, which includes receptor-positive tumours (oestrogen/progesterone and Her2neu) and triple negative breast cancer (TNBC). For the first group, targeted therapies have been developed and are currently widely used. On the contrary, for the second group, therapeutic options are extremely limited [10,11]. In the attempt to improve breast cancer patient outcome, several analyses have been performed not only to identify genetic mutations responsible for tumour onset, but also to find new putative therapeutic targets.

**Figure 2:**
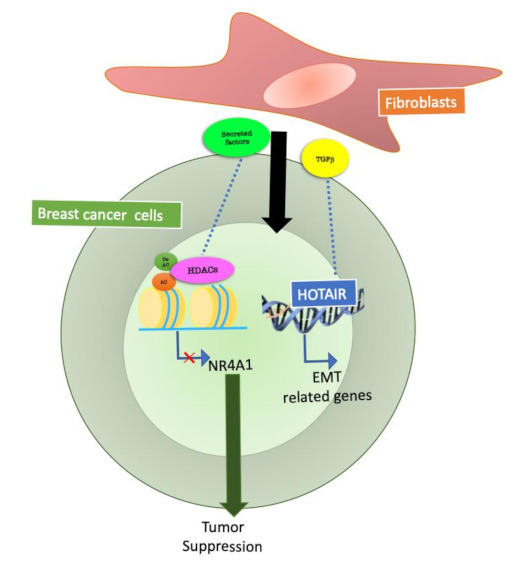
Epigenetic mechanisms at the basis of fibroblast communication with breast cancer cells. Fibroblasts are the most abundant stromal cells in breast cancer, and several studies have focused on fibroblasts and cancer cell communication. Fibroblasts, through secreted factors, can regulate HDACs (Histone deacetylases) and non-coding RNA activity in breast cancer cells and modulate tumour cell behavior.

In addition to the genetic mutations identified in BC, considerable attention has been given to epigenetic events in cancer cells [12]. It is important to notice that for all BC patients, metastasis to bone, brain, lungs and lymph nodes are the main cause of death, rather than the primary tumour [13]. Recent advances have well established that in order to invade and metastasize, breast cancer cells lean on systemic and local stromal interaction, and several studies have demonstrated the fundamental role of TME in both receptor positive and triple negative breast cancer [14]. TME/tumour crosstalk has been widely demonstrated, however the exact mechanisms by which this communication occurs are largely unknown. Certainly, the epigenetic modifications are strong candidates for such tasks. Thus, epigenetic changes has been investigated in order to explain tumour–stroma crosstalk in BC. Among the components of BC TME, cancer-associated fibroblasts (CAF) are the most abundant cell types, and support key processes such as proliferation, invasion, angiogenesis and resistance to treatment [15]. It has been suggested that, in order to support cancer progression, CAF may manipulate cancer cell gene expression through epigenetic mechanisms (Figure 2).

In 2018, Ren et al. focused on the epigenetic changes mediated by CAF on BC cells. The authors reported that transforming growth factor beta (TGFβ), secreted by CAF, is essential for epithelial to mesenchymal transition (EMT) and metastasis of breast cancer cells. TGFβ enhances the expression of the long noncoding RNA, HOX transcript antisense RNA (HOTAIR) in tumour cells. HOTAIR is a molecule that synergizes with chromatin-modifying enzymes with the aim of promoting the epigenetic regulation of gene expression [16].

Inhibition of TGFβ signals by small molecules in CAF reduces HOTAIR activation in cancer cells. These data were confirmed in a BC orthotopic mouse model in which HOTAIR depletion inhibits CAF mediated tumour growth and metastasis [17].

The class IIa Histone Deacetylase 7 (HDAC7) is an important epigenetic regulator expressed in breast epithelial cells [18]. HDAC7 can influence H3K27ac levels at super-enhancers in breast cancer stem cells [19]. HDAC7 levels were increased in Ras-transformed cells, in which this protein was required for cancer stem-like cell growth and invasive behavior by sculpturing the tumour microenvironment [20]. The effects of class IIa HDACs on Er+ cancer cells proliferation can also be explained by the repression of NR4A1/Nur77 operated by these enzymes [21]. This mechanism is likely to be sustained by fibroblasts. A recent work demonstrated that PIK3Cδ expressed in fibroblasts is involved in fibroblast-mediated TNBC cell invasion. Inhibition of f-PIK3Cδ, on the other hand, promoted the secretion of factors that led to the upregulation of NR4A1 in TNBC cells [22]. This suggests that fibroblasts could mediate the activation of class IIa HDACs in BCs, resulting in NR4A1 modulation, ultimately influencing invasion and proliferation of tumour cells.

In addition to these cell-based data, recent pre-clinical and clinical studies have reported the efficacy of an epigenetic therapy in unsettling the premetastatic niche.

Myeloid derived suppressor cells (MDSCs) are cells tight related to neutrophils and monocytes. In a healthy organism, this cell type is absent, while MDSCs are detectable in pathological conditions such as cancer and inflammation. Recently published evidence highlighted MDSCs as an important player in supporting tumour progression [23]. In particular, Lu et al. have shown that after removal of primary breast, lung and oesophageal cancer, adjuvant therapy with low doses of methyltransferase inhibitors (5-azacytidine) and histone deacetylase inhibitor (entinostat) inhibits the trafficking of myeloid-derived suppressor cells (MDSC), by promoting their differentiation into a more macrophage-like phenotype. It is important to highlight that the inhibition of HDACs in this setting could also affect fibroblasts in the metastatic niche, as entinostat has been administrated systemically [24].

In a work recently published, the “ChromSCape” application was used to study the epigenome of mouse breast tumour TME. Epigenomic fluctuations were identified within the tumour microenvironment, highlighting a distinct H3K27me3 landscape associated with cell identity and breast tumour subtypes [25].

In summary, in breast cancer, epigenetic mechanisms play crucial roles also in tumour stroma crosstalk, and future and current ongoing studies will further elucidate these mechanisms to eventually translate these findings into clinical practice.

### 2.2. Ovarian Cancer

Ovarian cancer (OC) is a serious health problem worldwide. In the majority of the cases, tumours are diagnosed at a late stage, when cancer cells have already spread to metastatic sites [26].

About 80% of the patients with advanced OC respond to the first-line treatment (surgery and platinum-based chemotherapy), however lasting diseases will eventually progress into chemo-resistant ovarian cancer and relapse within 16–22 months occurs in the majority of cases [27]. For this reason, the 5-year survival rate of patients with stage III and IV ovarian cancer remains extremely low [28].

OC cells quickly colonize the peritoneal cavity, building a complex tumour microenvironment (TME) that supports ovarian cancer survival, growth and spread [29,30,31].

**Figure 3:**
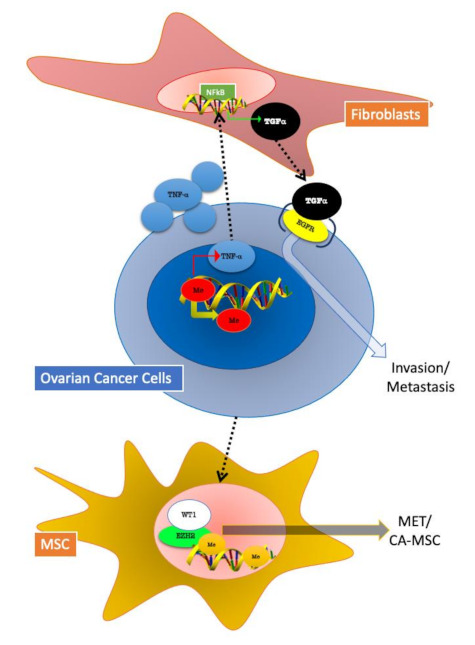
Epigenetic changes involved in tumour–stroma crosstalk in ovarian cancer (OC) cells. Fibroblasts and MSC (mesenchymal stem cells) can both influence and be influenced by OC cells. In both represented mechanisms, epigenetic modifications play a pivotal role. Fibroblasts in OC TME are involved in a TNFα/TGF-α/EGFR (epidermal growth factor receptor) loop activated by DNA hypomethylation, which sustains cancer cells invasion and metastasis. OC cells mediate mesenchymal to epithelial transition (MET) in MSC by the activation of the epigenetic regulator EZH2 (enhancer of zeste homolog 2).

In a work published in 2017, it was investigated how cancer epigenetics may regulate tumour–stromal interactions in peritoneal metastasis of OC. Researchers found that stromal fibroblasts enhance colony formation of metastatic OC cells. Furthermore, expression of transforming growth factor-alpha (TGFα) is induced in stromal fibroblasts co-cultured with OC cells. In addition, the authors observed an over-expression of tumour necrosis factor-alpha (TNFα) in ovarian cancer cells, regulated by DNA hypomethylation in its promoter, as well as by chromatin remodelling. Finally, OC-derived TNF-α induced TGFα transcription in stromal fibroblasts through nuclear factor-κB (NF-κB). On the other hand, TGFα secreted by stromal fibroblasts in turn promotes peritoneal metastasis of ovarian cancer, through epidermal growth factor receptor (EGFR) signalling. In summary, this research group identified a loop between the tumour and stromal component of metastases, formed by TNFα-TGFα-EGFR. These results suggest that cancer epigenetics may induce a loop of the cancer–stroma–cancer interaction that may promote peritoneal metastasis of OC cells via TNFα-TGFα-EGFR [32] (Figure 3).

Further efforts have been made to determine the role of TME in OC. In the 2011, a critical stromal stem cell, the carcinoma-associated mesenchymal stem/stromal cell (CA-MSC) was identified [33]. These cell types arise from resident tissue normal mesenchymal stem cells. [34]. Most importantly, CA-MSCs, have normal genomes and lack tumour-related mutations and malignant potential [35]. It has been demonstrated that OC cells growth is enhanced by CA-MSC; furthermore, this cell type is able to mediate OC resistance to therapy. CA-MSCs are able to increase cancer cell stemness and modify the surrounding TME by enhancing tumour fibrosis and promoting angiogenesis [36]. Very recently, Fan et al. demonstrated that CA-MSCs are characterized by enhancer-enriched DNA hypermethylation, increased repressive histone modifications and altered chromatin accessibility. The authors identified a cancer-mediated epigenetic reprogramming of MSCs intended to form the cancer-supportive CA-MSC. Unpredictably, this reprogramming induced a partial mesenchymal-to-epithelial transition (MET) that boosted cell contact pathways between CA-MSCs and tumour cells. This binding was found to increase OC metastasis. Enhancer of zeste homolog 2 (EZH2) and Wilms’ tumour protein (WT1) were identified as potential mediators of CA-MSC epigenetic MET reprogramming. Thus, treatment with an EZH2 inhibitor decreases OC metastatic potential in an orthotopic mouse model. Contrary to tumour cell epithelial-to-mesenchymal transition (EMT), stromal MET appears to be important for ovarian cancer in order to form metastasis, and MET may be a promising target for future therapy design [37] (Figure 3).

In conclusion, several components of the OC TME have been found to promote cancer trough epigenetic mechanisms. In these tumour types, targeting tumour–stroma crosstalk trough epigenetic mechanisms may be fundamental in challenging the inception of metastasis.

### 2.3. Colorectal Cancer

Colorectal cancer (CRC) is among the leading cause for cancer-associated death worldwide [38]. Several genetic alterations have been reported in CRC patients, mainly related to the Wnt pathway. However, emerging evidence suggest that non-genetic factors, such as the TME can support oncogenic transformation in CRC. It has been demonstrated that Wnt pathway activity is increased in cancer cells that grow close to myofibroblasts, supporting the importance of TME in CRC [39]. The importance of events beyond genetic mutations have been highlighted in these malignancies too. Thus, also in CRC, epigenetic mechanisms can be tumour–stroma crosstalk.

In 2018, Agrawal et al. demonstrated that fibroblasts accelerate cell proliferation and differentially control the expression of DNA methylation-regulating enzymes, enhancing Decitabine-induced demethylation in CRC cells. In contrast, conditioned medium from senescent fibroblasts, by upregulating NF-κB activity, altered deoxycytidine kinase levels in drug-untreated CRC cells and abrogated the Decitabine effect on the degradation of DNA methyltransferase. The authors investigated the effects of senescent fibroblasts in a 3D culture of CRC cells. They found that senescent fibroblasts increased DNA demethylation of CRC spheroid cultures and increased their stemness [40].

**Figure 4:**
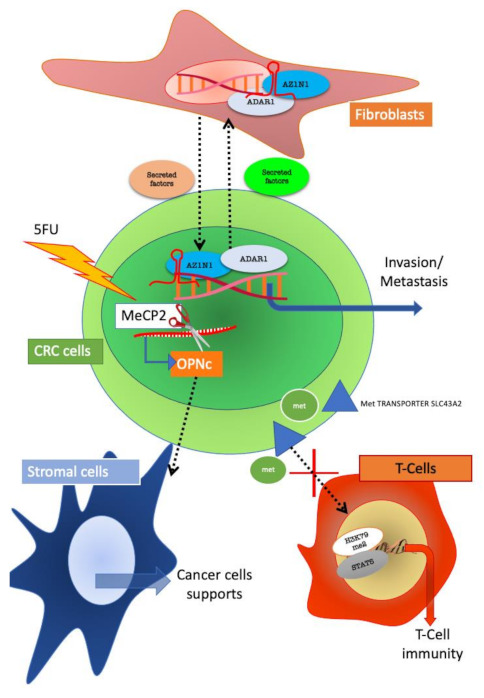
Reciprocal influences of colorectal cancer (CRC) and stroma cells mediated by epigenetic mechanism. CRC and fibroblasts interact through the ADAR1/AZIN1 (Adenosine deaminase acting on RNA 1/Antizyme Inhibitor 1) system and through this mechanism support tumour progression. In CRC resistant to 5FU, the isoform C of osteopontin (OPN) is selectively expressed; the isoform is produced by alternative splicing mediated by MeCP2 (methyl CpG binding protein 2). Secreted OPNc influences surrounding stromal cells towards sustaining tumour growth. CRC cells can also impede T-cell immunity by methionine subtraction which leads to H3K79m2/STAT5 inactivation in T cells.

Other groups focused on the role of fibroblast in CRC. A-to-I RNA editing (ADAR) is a recently described epigenetic modification that may contribute to human cancer onset. In 2018, a research group analysed a cohort of 627 colorectal cancer (CRC) samples and investigated the expression pattern of ADAR1 and its effects on? antizyme inhibitor 1 (AZIN1) mRNA levels. Both ADAR1 and AZIN1 expression was found to be significantly increased in CRC tissues as compared to normal tissue. Intriguingly, ADAR1 expression was specifically upregulated in both cancer cells and fibroblasts from tumour tissue. Conditioned medium from cancer cells induced ADAR1 expression and activation of AZIN1 in fibroblasts. On the other hand, edited AZIN1 enhanced the invasive potential of fibroblasts. The ADAR1–AZIN1 system represents a way of tumour–stroma crosstalk communication, which is able to influence both tumour and stromal cells behaviour [41] (Figure 4).

Epigenetic mechanisms are operative also in stroma-mediated resistance to treatment.

Resistance to 5-fluorouracil (5-FU) chemotherapy in CRC is still an unresolved challenge [42]. The role for secreted factors in promoting drug resistance has been widely demonstrated. Osteopontin (OPN) is a secreted protein involved in several pathological mechanisms including inflammation and cancer [43].

In colon cancer cells, over expression of OPN has been detected [43]. A recent study reported that OPNc is the most increased splicing isoform of OPN after 5-FU treatment of colon cancer cells. The authors show that OPNc promoted cell survival in 5-FU more than any other OPN isoforms. Intriguingly, Methyl-CpG binding protein 2 (MeCP2) was found to be responsible for the drug-induced splicing regulation of the OPN gene. These results demonstrate that the production of OPNc is epigenetically regulated by MeCP2. Ultimately, OPNc secretion, could transmit the stress signals elicited by chemotherapy to the TME, thus favoring the survival of adjacent colon cancer cells [44].

The role of MDSC has been highlighted also in CRC. The expansion of MDSC is detectable in the circulation and tumour microenvironment of CRC. A recent study reported the transcriptomic profiles of tumour-infiltrating MDSCs in CRC patients and uncovered pathways, which could potentially assist tumour progression. The author further investigated different subsets of circulating MDSCs in CRC patients and investigated their transcriptomic profiles in order to disclose pathways, which could potentially contribute to disease progression. They identified different subtypes of MDSCs: polymorphonuclear/granulocytic MDSCs (PMN-MDSCs) and monocytic MDSCs (M-MDSCs). By evaluating gene expression of MDSC subtypes, the author identified among others, DNA damage pathways. Moreover, acetylation-related genes were upregulated in both PMN-MDSCs and M-MDSCs. This latter finding implicates that epigenetic modifications mediated by PMN-MDSCs and M-MDSCs could also play a role in the regulation of multiple tumour-promoting genes in CRC [45].

Fibroblasts and MDCSs are not the only components of the TME that can have a role in modulating cancer cell behavior. In 2020, a research group identified the importance of the environmental glutamine in a CRC patient. Environmental glutamine restriction was able to augment WNT signalling in APC-mutant intestinal organoids to promote cancer stemness. This mechanism led to adenocarcinoma formation in vivo via decreasing intracellular α-ketoglutarate (αKG) levels. αKG addition was sufficient to counteract low-glutamine-induced stemness and Wnt hyperactivation by promoting hypomethylation of DNA and histone H3K4me3, resulting in upregulation of differentiation-associated genes and downregulation of Wnt target genes. In CRC derived organoids and in in vivo CRC tumour models, αKG addition decreased Wnt signalling and promoted cellular differentiation, resulting in a significant decrease of tumour growth [46].

In addition to the above-described mechanisms of epigenetic modulation of tumour–stroma crosstalk, a recent evidence suggests that also immune cells have the ability to influence tumour growth by epigenetic mechanisms.

Epigenetic patterns have been associated with the role of T cells in tumour [47,48]. A recent study shows that CRC, melanoma and ovarian tumour cells disrupt methionine metabolism in CD8+ T cells, thereby lowering intracellular levels of methionine and the methyl donor Sadenosylmethionine (SAM). Lower methionine availability resulted in loss of demethylation at lysine 79 of histone H3 (H3K79me2). The loss of H3K79me2 leads to decreased expression of STAT5 and impaired T-cell immunity. Tumour cells consumed methionine and “starving” T cells for methionine by expressing high levels of the methionine transporter SLC43A2. Genetic and biochemical inhibition of tumour SLC43A2 restored H3K79me2 in T cells and re-established tumour immunity. In addition, methionine supplementation improved the expression of H3K79me2 and STAT5 in T cells, coupled with the increase in T-cell immunity in mouse models and CRC patients. From a clinical point of view, tumour SLC43A2 correlated negatively with T-cell histone methylation [49].

In summary, many studies have identified the role of epigenetics in tumour–stroma crosstalk in CRC. Fibroblast, MDSC, immune cells and the metabolic microenvironment communicate with colorectal cancer to be reciprocally influenced. Through epigenetic modifications, the genome is functionally reorganized in order to sustain an aggressive proliferative phenotype.

### 2.4. Pancreatic Cancer

Pancreatic carcinoma (PC) is one of the most lethal and aggressive malignancies, with an extremely low 5-year survival rate [50]. The lack of early symptoms and aggressive biological characteristics of tumour are the reasons why only 15–20% of patients are diagnosed at early stage when tumours are still resectable [51].

**Figure 5:**
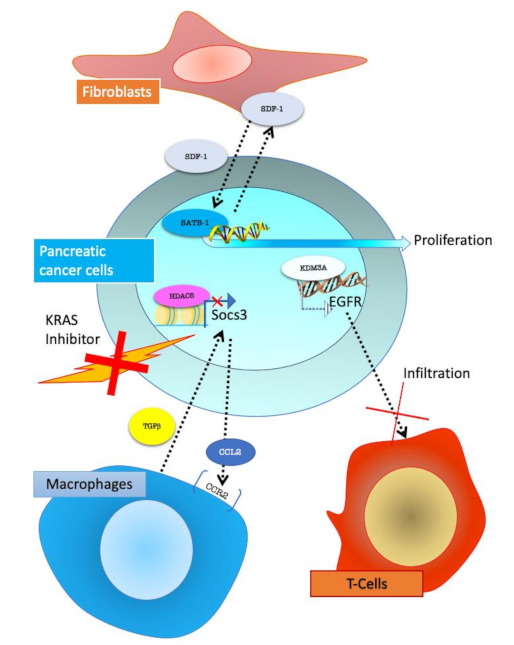
Pancreatic cancer cells interactions with stroma cells. Fibroblasts, through SDF-1 (stromal cell-derived factor 1) secretion, activate SATB-1 (special AT-rich sequence-binding protein-1) epigenetic regulator in PC cells promoting proliferation. In KRAS inhibitor-resistant PC cells, HDAC5 (Histone Deacetylase 5) through gene expression modulation, promotes CCL2 (C-C Motif Chemokine Ligand 2) secretion, which allows the recruitment of CCR2+ (C-C Motif Chemokine Receptor 2 positive) macrophages. Macrophages can secrete TGFβ (transforming growth factor beta 1) and support tumour growth. KDM3A (lysine-specific demethylase 3A) in cancer cells regulated epigenetic EGFR expression which decreases T-cell infiltration.

PC is characterized by profuse surrounding tissue which accounts for about 80% of tumour mass. This feature forms the TME where CAFs are the most abundant stromal cell type [52]. Thus, in PC, fibroblasts play a fundamental role, and many studies have investigated their role in sustaining tumour progression [53]. Recently several research groups have highlighted epigenetic mechanisms in CAF/tumour interactions. Wei et al. in 2018 investigated the role of SATB-1 (Special AT-rich sequence-binding protein 1) in this setting. SATB-1 can fasten genomic loci to the nuclear matrix to form high-order chromatin structures. It also recruits multiple chromatin-modifying enzymes and transcription factors to regulate global gene expression by modifying histones and remodelling nucleosomes. Increasing evidence indicate that SATB-1 upregulation is associated with poor prognosis in prostate and breast cancer [54,55]. Wei and co-authors characterized the role of SDF-1, a chemokine secreted by CAF, in modulating SATB-1 in PC cells. SDF-1 upregulated the expression of SATB-1 and this upregulation supported tumour progression and resistance to treatment in PC cells. In turn, the overexpression of SATB-1 in PC cells preserved surrounding CAFs, suggesting a circuit formed by CAF SDF1 and SATB-1 in cancer cells that promotes PC progression. Importantly, the authors found a clinical correlation of SATB-1 and SDF-1 in human pancreatic cancer specimens [56]. These data further support the pivotal role of fibroblasts/tumour crosstalk and highlight the epigenetic mechanism at the basis of this process (Figure 5).

Despite CAFs representing the most abundant cell types in PC TME, immune cells have also been described as influencing not only tumour progression, but also response to treatment in PC [57,58].

One of the main challenges of PC management is the intrinsic and acquired resistance to currently employed treatment. Immunotherapy has recently been introduced as a new therapeutic tool. Disappointingly, PC does not respond to this treatment, and this failure has been related to poor T-cell infiltration in the TME [59].

In a work published in 2020, an in vivo CRISPR screening, performed using mouse clonal congenic primary tumour cell lines, identified lysine demethylase 3A (KDM3A) as a potent epigenetic regulator of immunotherapy response in PC. The authors show that KDM3A acts through Krueppel-like factor 5 (KLF5) and SMAD4 to regulate the expression of EGFR. Knock-down of KDM3A, KLF5, SMAD4 or EGFR in tumour cells influenced the immune TME and re-sensitized tumours to combination immunotherapy. Practically, treatment of established tumours with an EGFR inhibitor erlotinib prompted a dose-dependent increase in intratumoural T cells [60].

Epigenetics may play a role in TME-mediated acquired resistance to therapy. Recent research reported an epigenetic mechanism involving TME behind PC cell resistance to a Ras inhibitor.

Kras mutations occur in more than 90% of PC cases [61]; constitutive activation of KrasG12D drives unrestrained proliferation and boosts survival of cancer cells [62], thus several strategies have been developed in order to inhibit the KRAS signalling pathway [63]. However, resistance mechanisms have been often described [64].

Very recently, the mechanisms involved in TME mediated resistance to treatment targeting oncogenic KRAS has been investigated. A gain-of-function screen of epigenetic regulators, performed in a KrasG12D p53 null PC mouse model, identified HDAC5 as the top hit, enabling oncogenic KRAS-independent tumour growth.

Tumours that escape treatment trough HDAC5, showed a prominent neutrophil-to-macrophage phenotype as compared to oncogenic KRAS-driven tumours. The authors described a role of HDAC5 in repressing Socs3 (Suppressor of Cytokine Signalling 3), which negatively regulates CCL2 (C-C Motif Chemokine Ligand 2). Repression of Socs3 due to HDAC5 action resulted in increased CCL2 and the consequent recruitment of CCR2+ (C-C chemokine receptor type 2) macrophages. Consistently, increased Ccl2 promoted macrophage recruitment into the TME and enabled tumour recurrence following oncogenic KRAS extinction. The tumour-associated macrophages (TAMs) provided cancer cells with trophic support, including TGF-β, to enable oncogenic KRAS bypass in a Smad4-dependent manner [65] (Figure 5).

In addition to a dismal prognosis, the majority of PC displays intrinsic or acquired drug resistance, which further complicates patient management. Importantly, in PC the microenvironment component accounts for a large part of tumour mass. Thus, particularly in these malignancies, the role of TME must be elucidated. Recently published works have highlighted the crucial role of epigenetics in tumour–stroma crosstalk in PC.

The possibility of exploiting these mechanisms to develop target therapy for PC is a promising research field that deserved further effort.

## 3. Discussion

Recently, many studies have scrutinized the relationship between cancer cells and the surrounding stroma [66]. The role of TME in supporting tumour on set, progression and resistance to therapy has been widely confirmed in many different tumour types. However, the exact mechanisms by which cancer and stromal cells influenced each other are yet to be defined. Adjacent cells may communicate with each other through different mechanisms. Secretion of a specific chemokine, such as TGF-β, is one of the most investigated mechanisms in tumour–stroma interaction [67,68,69] (Table 1). Active molecules may be released by different cell types, and they may target several other cells. It is plausible that the signalling triggered by secreted factors could eventually induce chromatin remodelling thus affecting back-and-forth communication between cancer and stroma.

However, despite intensive research effort in the field, the exact mechanism by which secreted factors actually triggered cellular response has not been fully elucidated. Several mechanisms may be involved in this pathway, including receptor activation and signal transduction, but recently it has been highlighted that epigenetics may play an important role in this setting, and epigenetic changes may be the key mechanism to support tumour–stroma crosstalk. Among TME components, fibroblasts play a pivotal role in many tumour types, and their functions in influencing tumour phenotypes have been widely investigated [70]. Recent studies have highlighted how the pro-tumoural activity of fibroblasts may be mediated by epigenetic mechanisms. On the other hand, signals from cancer cells may be fundamental to sustaining CAF features, and also in this case, the signal is in some cases triggered and activated by epigenetic mechanisms.

In all tumour types evaluated in this review, the epigenetic mechanisms at the basis of fibroblasts/cancer cell crosstalk have been investigated.

In breast cancer cells, HDACs and non-coding RNA (nc-RNA) can be modulated by fibroblasts’ secreted factors, thus modulating cancer cell gene expression and eventually tumour behavior. A communication loop, which implies epigenetic regulators has been identified between ovarian cancer cells and stromal fibroblasts. In this case, DNA hypomethylation plays a role in EGFR expression. While in CRC, the ADAR/AZIN1 systems is involved in back-and-forth communication between cancer cells and fibroblasts. SDF-1 secreted by fibroblasts activates SATB-1 in pancreatic cancer cells, which in turn sustains tumour progression.

Aside from CAF, an important role in TME is played by MDSCs. Myeloid-derived suppressor cells are a heterogeneous group of cells with the ability to suppress T-cell functions. The number of MDSCs increases during cancer [71].

In MDSCs subtypes detected in CRC, one of the prominent activated pathways include DNA damage and acetylation-related genes; however, it has been shown that the use of low doses 5-azacytidine (DNAmethyltransferase inhibitor) and of the histone deacetylase inhibitor (entinostat) inhibits the trafficking of myeloid-derived suppressor cells (MDSC) in breast, lung and oesophageal cancer. In the case of this cell type, the role of epigenetics in modulating MDSCs/cancer cell interactions has been already identified in several cancer types. The immune components of TME can also exploit epigenetic mechanisms to influence and be influenced by cancer cells. T cells and macrophages rely on epigenetic signalling for cell–cell communication within TME. T-cell infiltration is an important mechanism to impede tumour progression; however, cancer cells may exploit different mechanisms to delay T-cell immunity, melanoma and ovarian tumour cells, for example, by disrupting the methionine metabolism in T cells, by lowering intracellular levels of methionine. Alternatively, pancreatic cancer cells, through the KDM3A-mediated overexpression of EGFR decrease T-cell infiltration. In this case, pharmacological inhibition of EGFR, restored T-cell presence in tumour stroma. Other stromal cell types, e.g., macrophages and mesenchymal stem cells, have been shown to rely as well on epigenetic mechanisms to communicate with cancer cells.

## 4. Conclusions

In summary, tumour–stroma crosstalk is a pivotal mechanism for cancer onset and progression. Epigenetic mechanisms have been investigated as possible tools at the basis of these cell–cell communications. Further studies are needed to scrutinize this mechanism, but current available data strongly support this hypothesis. The reprogramming of the epigenome is important not only in stromal mediated tumour progression and onset, but also in TME-mediated drug resistance.

In the future, identification of the epigenetic changes induced by cellular interactions may be used as new therapeutic targets for current untreatable tumours with dismal prognosis.

## Figures and Tables

**Table 1:** Examples of the molecules involved in epigenetic mediated tumour–stroma crosstalk.

TME Componet	Molecules Involved	Epigenetic Mechanism/Modification	Cancer Types
Fibroblasts	TGF-β	HOTAIR	Breast Cancer [16]
		ADAR	Colorectal Cancer [41]
	SDF-1	SATB-1	Pancreatic Cancer [56]
	TGF-α	Methylation	Ovarian Cancer [32]
Immune cells	methionine	Methylation/SAM	Colorectal Cancer [49]
		KDM3A	Pancreatic Cancer [60]
	TGF-β	HDAC5	Pancreatic Cancer [65]
MSC		EZH2/WT1	Ovarian Cancer [37]
MDSCs		Acetylation	Colorectal Cancer [44]
Metabolic microenviroment	glutamine	Methylation	Colorectal Cancer [47]
